# Isolation of Anammoxosomes From the Aggregate Culture of *Ca*. Brocadia Sapporoensis and Assembly of Ladderane Liposomes

**DOI:** 10.1002/bit.29011

**Published:** 2025-05-07

**Authors:** Tomáš Podzimek, Terezie Cisarová, Michal Dvořák, Barbora Vokatá, Christina Karmann, Jaroslav Hanuš, Martin Balouch, Matěj Malý, Jana Hajšlová, Vojtěch Kouba, Jan Bartáček, František Štěpánek, Petra Lipovová

**Affiliations:** ^1^ Department of Biochemistry and Microbiology University of Chemistry and Technology Prague Prague Czechia; ^2^ Department of Chemical Engineering University of Chemistry and Technology Prague Prague Czechia; ^3^ Department of Water Technology and Environmental Engineering University of Chemistry and Technology Prague Prague Czechia; ^4^ Department of Food Analysis and Nutrition University of Chemistry and Technology Prague Prague Czechia

**Keywords:** anammox bacteria, anammoxosomes, artificial liposomes, *Ca*. Brocadia sapporoensis, ladderanes

## Abstract

Anammox bacteria wield an energy‐efficient nitrogen metabolism enveloped in anammoxosome organelle composed of unique ladderane lipids. Thus, waste anammox biomass seems to be an attractive target for the isolation of ladderanes and subsequent production of artificial vesicles for drug delivery. This study proposed a novel method to isolate ladderane‐rich anammoxosomes from aggregate mixed culture of *Ca*. Brocadia sapporoensis. Compared to conventional isolation protocols, the protocol was simplified by omitting the prepurification of anammox cells, replacing Percoll® with a sucrose gradient and prolonging the application of EDTA. This enhanced and simplified procedure efficiently removed EPS and other debris, thus yielding the layer of anammoxosomes as confirmed by control experiments and TEM. For the first time, the resulting ladderane isolates were used for the preparation of liposomes, both with and without the addition of pure dipalmitoylphosphatidylcholine (DPPC). Vesicles were successfully created, characterised by TEM and DLS, and anammox‐based ladderanes were incorporated into their walls. These liposomes had interesting functional properties such as increased colloid stability at elevated concentrations, meaning a reduced tendency to form aggregates compared to model liposomes made solely of DPPC. Overall, this study offers insights into converting waste anammox biomass into a valuable resource for drug delivery.

## Graphical abstract

Ladderane lipids in anammox bacteria can find use in artificial liposomes for drug delivery. This study developed the isolation of ladderane‐rich anammoxosome organelles from previously challenging aggregate mixed culture of *Ca*. Brocadia sapporoensis. The resulting ladderane isolates were used for the preparation of liposomes which had interesting functional properties.

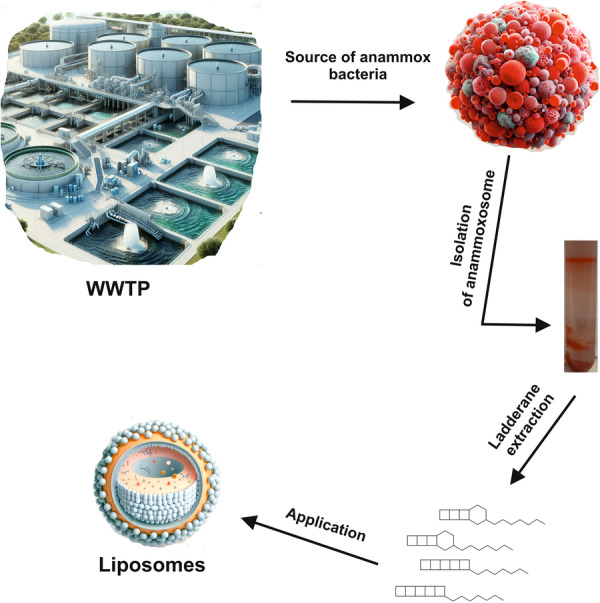

## 1 Introduction

Anammox bacteria are well known due to their ability to perform anaerobic oxidation of ammonia (Van Niftrik and Jetten 2012; Shaw et al. 2020). This process is utilized by wastewater treatment plants for the removal of nitrogen from wastewater (Wu et al. 2020; Mishra et al. 2022). Another interesting fact is that this bacterium possesses an organelle called anammoxosome, which is enveloped by a lipid bilayer. This organelle harbours enzymes of the central cell metabolism, including the conversion of nitrite and ammonia into a nitrogen molecule (Fuerst 2005). The membranes of anammox bacteria are composed of phospholipids, which in addition to “common” fatty acids, also contain ladderane structures (concatenated cyclobutane rings). These lipids were found to change the properties of the membrane to make it less permeable (Sinninghe Damsté et al. 2002; Boumann et al. 2009; Moss et al. 2018).

Unique permeability of anammox‐based ladderane lipids could be utilized in artificially prepared lipid vesicles serving as carriers for the delivery of different substances. In fact, liposomes are currently the most successful nanomedical devices. Since the FDA approval of the first liposome formulation, Doxil® (Barenholz 2012), numerous liposome formulations have been approved (Crommelin et al. 2020). Despite the great success of liposomes for several drugs, their potential is not fully realized due to the limited number of drugs that are compatible with liposome encapsulation (Balouch et al. 2021). This is mainly caused by premature leakage of cargo from liposome, which can be improved by changing the composition of phospholipid liposome membrane (Frallicciardi et al. 2022). Thus, new lipids that can be used as a liposome building block can improve the possibilities of liposome formulation for new candidate molecules.

For potential assembly of artificial liposomes, ladderane phospholipids can be obtained by the following approaches: 1) direct isolation from the anammoxosome membrane, 2) chemical synthesis of the entire ladderane phospholipid, and 3) insertion of the biosynthetic pathway into a suitable production organism. The first option includes direct extraction of ladderanes from disrupted biomass or the prior isolation of anammoxosomes enriched with ladderanes followed by the extraction of the lipids. The first release of anammoxosomes from bacterial cells was successfully performed using aggregated culture of *Candidatus* Brocadia anammoxidans (Sinninghe Damsté et al. 2002). However, they did not separate the anammoxosomes from the rest of the cell debris. The first separation of anammoxosomes was described twelve years later, when anammoxosomes were successfully isolated and separated from a planktonic culture of *Candidatus* Kuenenia stuttgartiensis (Neumann et al. 2014). The second approach to obtain ladderanes was achieved by Mascitti and Corey (2006), who synthesized pentacycloanammoxic acid, and later by Mercer et al. (2016), who published the synthesis of [5]‐ladderanoic acid and [3]‐ladderanol, which were assembled into a complete phospholipid. The synthesis of such a complex molecule involved many reaction steps, required a number of different reactants and catalysts, and provided a poor yield (Javidpour et al. 2016). The third option is under investigation and remains unclear. Rattray et al. (2009) proposed two hypothetical pathways for the biosynthesis of lipids by anammox bacteria. In 2016, Javidpour and his colleagues made an effort to introduce a lipid biosynthetic pathway into *E. coli*, unfortunately unsuccessfully (Javidpour et al. 2016). Hence, it can be speculated that the annotation of genes predicted to be involved in lipid biosynthesis in anammox bacteria is not correct.

Of these methods for obtaining ladderane isolates, the extraction of anammoxosomes followed by separation using HPLC seems to have the highest potential. One of the two challenges for effective isolation of anammoxosomes is the presence of an extracellular matrix containing extrapolymeric substances (EPS) in aggregated cultures. EPS include polysaccharides, proteins, DNA, inorganic compounds, etc., which surround individual cells (Nouha et al. 2018). The second factor is that a consortium of microorganisms naturally inhabits these aggregates, therefore anammox bacteria always coexist in a mixed culture which dilutes the ladderane lipid content (Guo et al. 2016). Thus, to obtain ladderane‐rich anammoxosome membranes for artificial liposomes from mixed and aggregated anammox cultures, it is crucial to optimize the anammoxosome isolation procedure, especially by enhancing the disruption and removal of EPS and other contaminant bacteria.

In this study, we describe a procedure for isolation and separation of anammoxosomes from mixed aggregate culture of *Ca*. Brocadia sapporoensis, including the description of microbial consortium and EPS. The novelty of this approach is that the procedure for isolating anammoxosomes from one type of anammox culture may not be applicable to another anammox culture. Moreover, in this study we attempt to incorporate a natural ladderane substrate extracted from anammox bacteria into artificially created liposome membranes.

## 2 Methodology

### 2.1 Material

Humic acid (cat. nr. H16752) used as a standard was purchased from Merck. Cellulase Onozuka R‐10 (cat. nr. C8001.0010) and Macerozyme R‐10 (cat. nr. C8002.0010) were purchased from Duchefa Biochemie. Proteinase K was purchased from Roche (cat. nr. 03115887001), α‐amylase from Merck (cat. nr. A6814‐1MU), β‐amylase from Merck (cat. nr. A7130‐10KU), and DNase I from Roche (cat. nr. 101041159001). 1,2‐Dipalmitoyl‐sn‐glycero‐3‐phosphocholine (DPPC) was purchased from Corden Pharma and 5(6)‐carboxyfluorescein (CF) from Merck.

### 2.2 Anammox Cultures

Both planktonic culture of *Ca*. Kuenenia stuttgartiensis and aggregated culture of *Ca*. Brocadia sapporoensis were kindly provided by Laura van Niftrik from Radboud University (Radboud University, Department of Microbiology, Institute for Water and Wetland Research). The culture of *Ca*. K. stuttgartiensis was used directly for the isolation of anammoxosomes, while *Ca*. B. sapporoensis was further cultivated as described below.

### 2.3 Cultivation of Anammox Bacteria

Aggregated biomass enriched in ‘*Ca*. B. sapporoensis’ was adopted as an inoculum of a cultivation reactor, initially originating from Radboud University. This fed‐batch reactor (FBR) with an effective volume of 8 L was operated for more than 900 days, at 30°C and a pH of 7.2. The anoxic conditions were maintained by sparging the liquid with a mixture of CO_2_/N_2_ (5%/95%).

Each operation cycle lasted 6 h consisting of reaction time (5.5 h), operation without influent (10 min), settling (10 min) and effluent draw (10 min). This cycle was performed with timers and CompactRIO® real‐time controller system and the application LabView (National Instruments). The FBR was fed with a synthetic substrate as shown in Table 1. Moreover, the substrate contained ammonium chloride and sodium nitrite in a molar ratio of 1:1. The concentrations varied 0,280–0,700 g‐N/L for ammonium chloride and 0,266–0,686 g‐N/L for sodium nitrite depending on the performance of the FBR.

**Table 1:** Composition of the mineral medium/feed to the reactors.

Compound	Concentration (mg.L^−1^)
Calcium chloride (CaCl_2_)	100
Magnesium sulphate (MgSO_4_)	300
Potassium hydrogen phosphate (KH_2_PO_4_)	30
Potassium hydrogen carbonate (KHCO_3_)	500
Iron sulphate (FeSO_4_.7H_2_O)	5
Hydrogen chloride (HCl)	1
Trace elements	1^a^, ^b^

^a^ Unit: mL.L^−1^.

^b^ Trace elements composition was taken from Van de Graaf et al. (1996).

The substrate and effluent of the reactors were regularly sampled to determine concentrations of NH_4_^+^‐N, N‐NO_3_^−^‐N, and NO_2_^−^‐N measured using Gallery™ Discrete Analyzer (Thermo Fisher Scientific) according to the standard methods (Apha 1998), the biomass concentration was measured as total suspended solids (TSS) and volatile suspended solids (VSS) according to standard methods (Apha 1998).

### 2.4 Amplicon Sequencing

Using the DNeasy Power Soil Kit (QIAGEN), DNA was extracted from isolated biomass following the manufacturer's instructions. Subsequently, the concentration of the extracted DNA was measured using the Qubit® dsDNA HS Assay Kit (Thermo Fisher Scientific). Furthermore, DNA purity and its concentration was measured using a nanospectrophotometer (BioDrop µLITE, BioDrop) and stored at −20°C.

For the commercial sequencing of the extracted DNA, libraries were prepared employing a two‐step PCR with Nextera technology. The target regions were the hypervariable regions V3 and V4 of the 16S rRNA gene, using the primers NEXTERA_357F (CCTACGGGNGGCWGCAG) and NEXTERA_805R (GACTACHVGGGTATCTAATCC). As a positive control, the ZymoBIOMICS microbial community DNA standard (Zymoresearch) was included. Sequencing of the libraries was performed on the NovaSeq. 6000 platform.

Fast QC software (v0.11.9) and MultiQC software (v1.9) were used for the evaluation of sequencing data quality. Cutadapt software (v1.18) was then used for clipping of adaptors, primers, and low‐quality bases (phred score < 25) (Martin 2011). Metataxonomic analyses were conducted through the DADA2 pipeline, using the Silva 138 database for taxonomy assignment (Quast et al. 2012).

Subsequent analysis involved the examination of amplicon sequence variants (ASVs) using the Phyloseq Package in R and sequences related to chloroplasts, mitochondria, or the eukaryotes were excluded (McMurdie and Holmes 2013). Similarly, ASVs with an abundance less than 30, comprising less than 3% of the data, were also discarded. Finally, the alignment of ASVs was performed using the R package DECIPHER (Wright 2016), and the resulting alignment was utilized to construct a maximum‐likelihood phylogenetic tree with IQ‐TREE 2 software (Minh et al. 2020).

### 2.5 Extraction and Analysis of Extrapolymeric Substances (EPS)

The aggregated culture of the reactor consisted of a mixture of granules and flocs. For EPS extraction, 0.2 g of lyophilized biomass was mixed with 15 mL of PBS buffer. After rehydration, the suspension was homogenized with Homogenizer D1000 (Benchmark Scientific) for 1 min at speed 2 (to avoid cell disintegration). Homogenized biomass was compared to the untreated biomass using a bino loupe at a magnification of 20×. The extract was separated from the cells by centrifugation at 9000 g for 10 min at 4°C.

The extract was analyzed for the presence of proteins, saccharides and humic acids. The phenol‐sulfuric acid method was used for the determination of total saccharides (DuBois et al. 1956). The content of both proteins and humic acids was determined with the combination of Lowry and modified Lowry method according to the publication of Frølund et al. (1995). Evaluation of proteins and humic acids was done using the equations:

$$A_{\text{PT}}=1.25\left(A_{L}–A_{\text{mL}}\right)$$

$$A_{\text{HA}}=A_{\text{mL}}–0,2A_{\text{PT}}$$

where A_PT_ is the absorbance corresponding to the amount of proteins, A_HA_ is the absorbance corresponding to the amount of humic acids, A_L_ is the overall absorbance obtained by the Lowry method, and A_mL_ is the overall absorbance obtained by the modified Lowry method (Frølund et al. 1995). The concentrations of the substances were determined using calibration curves. From these, the masses of the substances were calculated, and the masses were related to the mass of the lyophilized biomass, which was finally expressed as a percentage.

### 2.6 Disruption of EPS

Several approaches have been tested to disrupt EPS in the extracellular matrix and release cells. To degrade possible polysaccharides in the extracellular matrix, a mixture of Onozuka cellulase R‐10 and Macerozyme R‐10, both at a concentration of 4% (w/v), was applied to the aggregated culture. These glycosidases were chosen to cleave cellulose, hemicellulose and pectins. The mixture was shaken for 3 h under laboratory temperature on a rotary shaker F 205 (Falc Instruments).

To degrade various substrates in the extracellular matrix, a mixture of different hydrolytic enzymes was used (Table 2). The culture from the reactor was centrifuged at 10000 g for 15 min at 4°C and the pellet was resuspended in 5 ml of 100 mM HEPES buffer pH 6.8. The aggregates were previously sonicated at 9 W for 30 s on ice and the suspension was treated with a mixture of hydrolytic enzymes (Table 2) at 37°C and the time was extended up to 24 h.

**Table 2:** Hydrolytic enzymes and their composition in a mixture.

Enzyme	Concentration in the sample
Proteinase K	20 μg. mL^−1^
α‐amylase	2 mg. mL^−1^
β‐amylase	2 mg. mL^−1^
Cellulase	20 mg. mL^−1^
DNase I	20 μg. mL^−1^

EDTA was also used to release cells from the matrix. The culture was shaken with 10, 20 and 50 mM EDTA, respectively, for 3 h at room temperature on the rotary shaker F 205 (Falc Instruments).

All suspension were added on either 78%, 50%, 20% or 10% Percoll® gradient after incubation and centrifuged at 14000 g for 40 min at 10°C.

### 2.7 Isolation of Anammoxosomes

Anammoxosomes were isolated from both planktonic *Ca*. K. stuttgartiensis and aggregated culture of *Ca*. B. sapporoensis. For the isolation of anammoxosomes from *Ca*. K. stuttgartiensis, the protocol of Neumann et al. (2014) was used. It contained both the prepurification of cells and the separation of anammoxosomes in a 78% and 50% Percoll® gradient, respectively. The buffers included in this protocol were both 10 mM HEPES buffer with 250 mM sucrose, pH 6.8 and 20 mM TRIS‐HCl with 250 mM sucrose and 0.2 mM EDTA, pH 6.6 as isolation buffer. For the isolation of anammoxosomes from *Ca*. B. sapporoensis, the same protocol was used, and a new modified one was developed, which is further described. The aggregated culture was centrifuged at 9000 g for 15 min at 4°C. The cell pellet was washed with 15 mL of 20 mM acetate buffer with 60 mM EDTA, pH 5 and centrifuged again. The cell pellet was weighed and resuspended (vortexed) in the buffer in the ratio of 1 g of cells:1.2–2.4 ml of buffer (depending on the viscosity of the sample). Suspended aggregates were treated with D1000 Hand‐held Homogenizer (Benchmark Sci) for 20 s at speed 6 and the suspension was pulled three times through the syringe with a needle (dimensions 0,8 × 40 mm). Suspension treated in this way was mixed for ca. 18 h on the rotary shaker F 205 (Falc Instruments) at laboratory temperature. The next day, the suspension was aliquoted in 2 mL tubes containing 0.5 mm Zirconia/Silica beads. Ca. 0.5 g of beads were used for 600 µL of the sample. The cells were then disintegrated in a FastPrep 24 homogenizer (MP Biomedicals) for 60 s at 6 m s^−1^. The samples were then collected from the tubes and placed in a sucrose gradient composed of 80% to 20% sucrose (v/v) in a centrifuge tube. The sucrose was prepared by diluting 2.5 M stock solution with 10 mM HEPES buffer with 60 mM EDTA, pH 7.5. The samples were centrifuged at 40000 g for 15 min at 20°C. The red layer lying on the sucrose was collected by a syringe with a needle (dimensions 0,8 × 40 mm). Finally, sucrose was washed twice by diluting the collected sample ten times with 10 mM HEPES pH 7.5 and centrifuged at 14000 g for 5 min at 20°C. The sample was stored at 8°C. The HEPES buffer was adopted from the work of Neumann et al. (2014) as a buffer for the storage of anammoxosomes.

### 2.8 Transmission Electron Microscopy

Isolated anammoxosomes were incubated in freshly prepared 3% glutaraldehyde in 0.1 M cacodylate buffer (pH 7.4) for 30 min. The sample was then centrifuged and washed three times with 0.1 M cacodylate buffer. The sample was then fixed with 1% osmium tetroxide for 1 h. The increasing concentration of ethanol (30, 50, 70, 90, 95% and 100% ethanol) was used to dehydrate the sample. The sample was embedded in a fresh AGAR 100 epoxy resin (Agar Scientific, UK). Ultra‐thin sections (~70 nm) of cells were cut with a diamond knife on a Leica UC6 ultramicrotome (Leica Microsystems, Wetzlar, Germany), collected on Parlodion® coated microscopy grids, and subsequently contrasted using saturated solutions of uranyl acetate and lead citrate.

The samples were analyzed using JEOL JEM‐1010 transmission electron microscope (Jeol, Japan) operated at 80 kV, equipped with SIS Megaview III CCD camera. The images were processed using AnalySIS software suit (Olympus, Japan).

### 2.9 Detection of Ladderane Lipids

The biomass was collected from the reactor and centrifuged at 9000 g for 15 min at 4°C. Cells were disrupted using OneShot Cell Disruptor (Constant Systems Ltd.) at a pressure of 2.5 kbar. The sample was then lyophilized, shaken in the extraction solvent mixture (MeOH:DCM:10 mM ammonium acetate, 2:1:0.8, v/v/v), sonicated, centrifuged, filtered and analyzed by the Dionex UltiMate 3000 RS ultrahigh performance liquid chromatography (U‐HPLC) system (Thermo Fischer Scientific, Waltham, USA), coupled to quadrupole‐time‐of‐flight SCIEX TripleTOF® 6600 mass spectrometer (SCIEX, Concord, Ontario, Canada) according to Kouba et al. (2022). The resulting data was processed according to Hurkova et al. (2019).

### 2.10 Preparation of Liposomes Containing Ladderanes

Liposomes were created using the Bangham lipidic film hydration method (Bangham et al. 1965). The lyophilized samples of the extracted lipids were dissolved in three cycles. In each cycle, the substrate was suspended/dissolved in a solvent (approximately 3 mg of sample per 1 ml of solvent), vortexed on a vortex shaker for 30 s, placed on a sonication probe and then centrifuged for 10 min under 12100 g at room temperature. At the end of each cycle, the supernatant was separated from the pellet, filtered with a syringe with a 200 µm filter and put into flask (inside the same flask after each cycle). The pellet was then resuspended with a new solvent in the next cycle. In each cycle, the polarity of the solvent decreased as the proportion of CH_3_OH:CH_2_Cl_2_ changed (1st cycle pure CH_3_OH, 2nd cycle CH_3_OH:CH_2_Cl_2_ 1:1, 3rd cycle CH_3_OH:CH_2_Cl_2_ 1:100). Remaining pellet after the third cycle was then dried under nitrogen stream and weighed. The measured pellet weight (typically < 10% of the initial extracted lipids weight chosen for each constructed mixture) was then subtracted from the initial extracted lipids weight so that the calculated ratio between DPPC and extracted lipids is based on the weight of dissolved lipids only.

The resulting solution (L9) was used for the creation of artificial ladderane‐containing vesicles. Vesicles containing only the solution of extracted lipids were prepared as well as mixed vesicles, containing extracted lipids with synthetic DPPC (see Table 3).

**Table 3:** Composition of artificially prepared phospholipidic vesicles.

	Dissolved L9 weight	DPPC weight	L9 percentage	
Sample name	(mg)	(mg)	(% w/w)	Hydration medium
L9‐100	4.68	0	100	TRIS‐HCl
L9‐76	8.17	2.64	75.6	TRIS‐HCl
L9‐29	3.33	8.02	29.3	TRIS‐HCl
DPPC	0	10.0	0	TRIS‐HCl
L9CF‐100	4.25	0	100	CF in TRIS‐HCl
L9CF‐74	4.85	1.7	74.1	CF in TRIS‐HCl
L9CF‐31	3.24	7.08	31	CF in TRIS‐HCl
CF: 5(6)‐Carboxyfluorescein				

Phospholipid solutions were placed on a rotary evaporator and the solvent was evaporated. All evaporation was performed on the rotary evaporator (Buchi Rotavapor R‐100). The phospholipid content dissolved in solvent was heated in the evaporator at 60°C. The pressure gradually decreased from atmospheric pressure until all the solvent evaporated (from 30 to 45 min reaching a pressure of 150 to 400 mbar, depending on the sample). After each evaporation, the film was placed into a desiccator for at least 6 h.

The resulting thin film was hydrated using TRIS‐HCl (25 mM), pH 8.5 or CF solution in 25 mM TRIS‐HCl, pH 8.5, and the final sample concentration was 3 mg of lyophilized sample per 1 ml of hydration medium. Hydration was carried out at 60°C for 1 h while constantly shaking the suspension. The suspension was then extruded using Avanti Mini Extruder, using 400 nm pore membranes. For observation of CF release from the vesicles, free CF was removed from the solution using PD MiniTrap G‐25 columns (Merck) at low temperatures (~10°C). The column was cooled with packed ice to maintain the temperature inside the device below 10°C.

The obtained vesicles were characterised using TEM, DLS, and confocal microscopy, and the release of entrapped carboxyfluorescein was studied using fluorescence spectroscopy. The microscope JEOL JEM‐1010 with an accelerating voltage of 80 kV was used for all the specimen TEM observation, mainly for the size and shape characterisation of the vesicles. The procedure proceeded as follows; First, 12 µL of the sample was placed on a carbon‐coated mesh grid and kept for free adhesion for 15 min. The residual sample that did not adhere or evaporate was then removed and the sample on a mesh grid was stained with uranyl acetate for 5 min.

DLS measurements were performed on Malvern Zetasizer Nano ZS in backscattering (173°) mode. Samples were measured in a 1 mL cuvette, diluted with deionized water. Typically, 30 µl of hydrated sample was added to 1 ml of deionized water.

Fluorescence spectrophotometer Cary Eclipse was used for the observation of the fluorescence of carboxyfluorescein loaded samples. Each time, 20 µl of the sample was added to 3 mL of TRIS‐HCl, pH 8.5. The excitation wavelength was set to 495 nm, and the excitation wavelength was set to 517 nm. Temperature ramps were used to assess the dependence of carboxyfluorescein release from vesicles on temperature. The sample was gradually heated (2°C/min) from 10°C up to 60°C and during that, the fluorescence was measured. After the end of the experiment, a drop of TRITON X‐100 was added to the sample to release all carboxyfluorescein from the vesicles. Then, the amount of carboxyfluorescein released was calculated as follows: the fluorescence of the sample at each temperature was divided by the fluorescence value of the Triton treated sample at the same temperature.

Fluorescence microscope Olympus Fluoview FV1000 with the 60x oil immersion objective was used for all microscopy observations. Each time, a 15 µl drop was observed. The excitation laser with 488 nm wavelength was used for sample illumination, and the emitted fluorescence was observed in a range from 505 to 540 nm.

## 3 Results and Discussion

### 3.1 Cultivation of Culture Containing Anammox Bacteria

**Figure 1:**
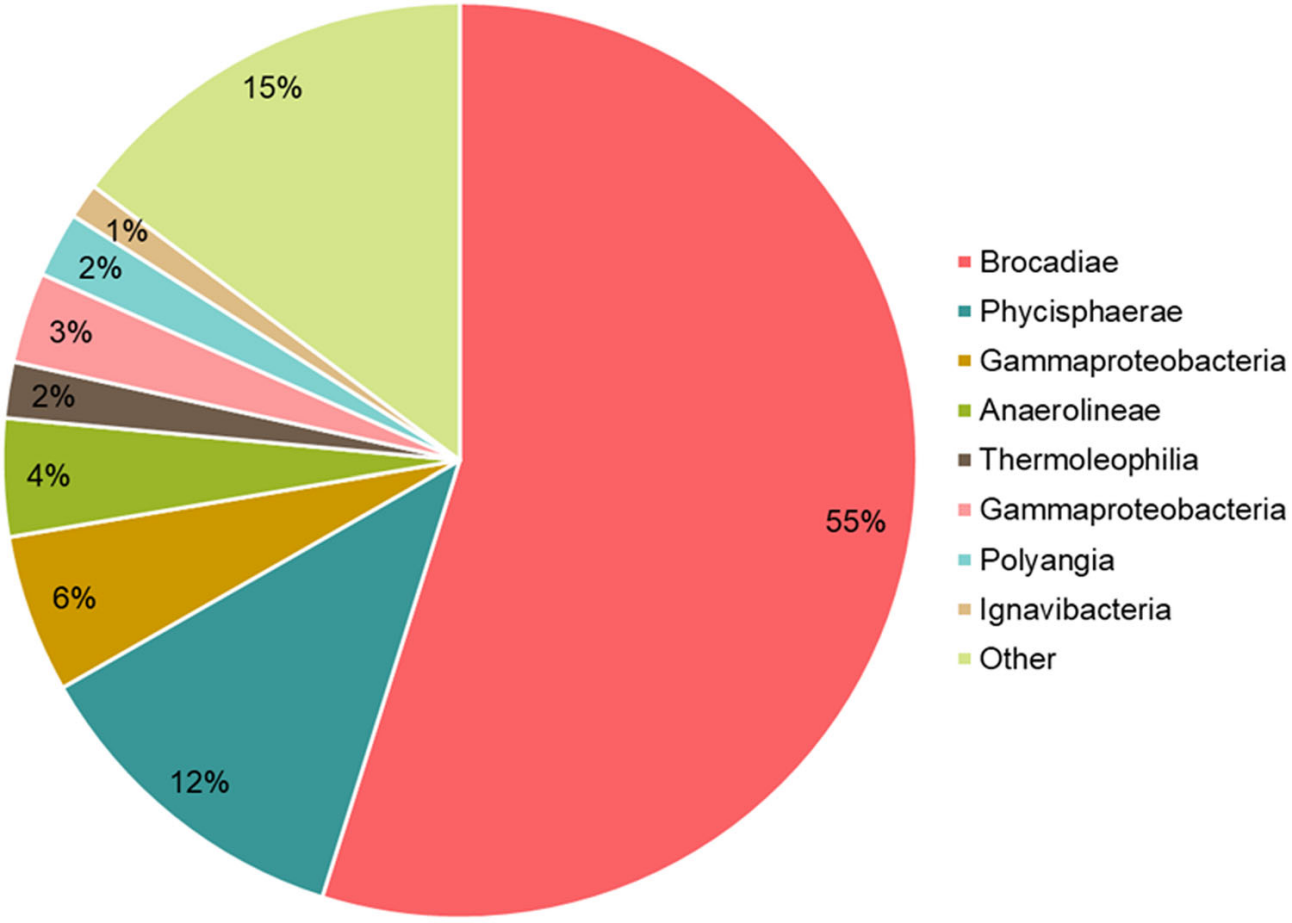
The classes of microorganisms identified in the biomass sample used for the extraction of anammoxosomes.

The cultivation reactor was operated for more than 500 days, with biomass consisting mainly of dark red granules that transformed into flocs over time and sometimes aggregating at the surface or walls of the reactor. The growth rate (μ_max_) was 0.01 d^−1^, lower compared to other bacteria, but similar to other studies on anammox bacteria (Van Der Star et al. 2008; Lotti et al. 2014; Laureni et al. 2015). The nitrogen loading ranged between 0.5 and 2.4 kg N m^−3^ d^−1^. Similarly, comparable to an optimal nitrogen loading range 0.7 to 2.0 kg N m^−3^ d^−1^ found in other studies (Tang et al. 2010; Yang et al. 2018; Dapena‐Mora et al. 2004). The amplicon sequencing showed the enrichment of *Ca*. Brocadia was found to be 55 ± 5% related to other organisms (shown in Figure 1). While an enrichment of over 90% of *Ca*. Brocadia was achieved in an MBR with a planktonic biomass for physiological characterization (Narita et al. 2017), other studies considered enrichments, even cultures with 15%–32% of anammox were considered enrichments (Awata et al. 2021; Connan et al. 2016). A similar abundance, ca. 50%, to the one observed in this study (55%) was achieved by other studies aiming to characterize anammox bacteria or develop kinetic models (Araujo et al. 2011; Pradhan et al. 2020).

Furthermore, it revealed a diverse microbial community with obligate anaerobes from the classes *Anaerolineae* and the *Ignavibacteria* and genus *Denitratisoma* from the class *Gammaproteobacteria*, associated with anaerobic environments in agreement with the conditions used for biomass cultivation in this study (Ribeiro et al. 2022; Nunoura et al. 2013; Chen et al. 2023; Iino et al. 2010).

### 3.2 Isolation of Anammoxosomes Using the Protocol for *Ca*. K. Stuttgartiensis

**Figure 2:**
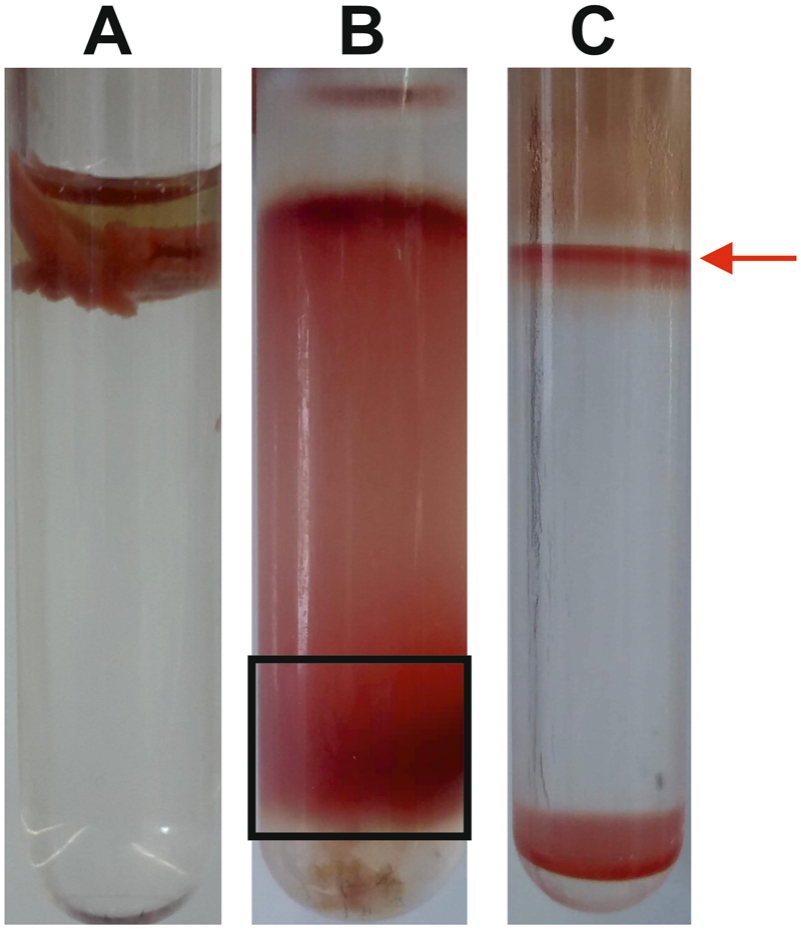
(A) Prepurification of aggregated *Ca*. (B). sapporoensis cells on a 78% Percoll® gradient. B) Prepurification of planktonic *Ca*. K. stuttgartiensis cells on a 78% Percoll® gradient (located in the black rectangle). (C) Anammoxosome layer (indicated by a red arrow) obtained from *Ca*. K. stuttgartiensis separated in a 50% Percoll® gradient according to Neumann et al. (2014).

The anammoxosomes were isolated from both *Ca*. Kuenenia stuttgartiensis and *Ca*. B. sapporoensis according to the procedure developed by Neumann et al. (2014). This procedure has been used successfully on a planktonic culture of *Ca*. Kuenenia stuttgartiensis. Therefore, in the present work, an isolation from *Ca*. Kuenenia stuttgartiensis was used as a control. In the first step, anammox cells are “prepurified” of other contaminating organisms. Because anammox cells have higher density, they sink to the bottom of the 78% Percoll® gradient (Figure 2B).

Efforts to prepurify *Ca*. B. sapporoensis cells resulted in the gluing of the entire biomass to a single clump that could not be separated in any way (Figure 2A). In the case of *Ca*. Kuenenia stuttgartiensis cells, prepurification was achieved by having the cells at a higher density and thus falling towards the bottom of the gradient (Figure 2B, black rectangle). The second step includes the isolation and separation of anammoxosomes in a 50% Percoll® gradient. The successful separation of *Ca*. Kuenenia anammoxosomes is shown in Figure 2C, where the organelles are present in a well‐defined layer on the top of the gradient (depicted by a red arrow).

To avoid the biomass sticking in the case of *Ca*. B. sapporoensis, we proceeded to characterize EPS and then select a cocktail of hydrolytic enzymes that could disrupt the interactions in the extracellular matrix, which could help to release the cells from aggregates.

### 3.3 Extraction of EPS

**Figure 3:**
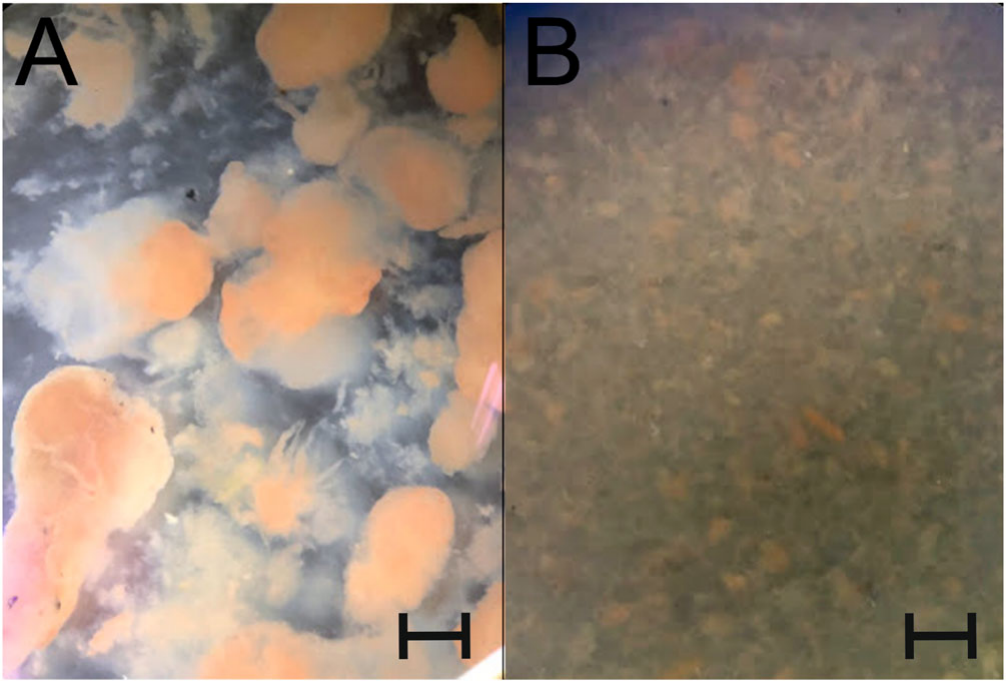
Example of aggregate breakdown. (A) culture before homogenization, (B) culture after homogenization using a homogenizer for 1 min at speed 2. Both samples were observed by bino loupe at a magnification of 20×. The scalebar represents 1 mm.

The extracellular matrix of aggregates, composed of various EPS, can pose a significant obstacle when attempting to isolate individual cells for subsequent experiments. To address this challenge, it is essential to have a good understanding of the composition of the extracellular matrix. In this study, we determined the composition of the main substances that usually form the extracellular matrix of bacterial aggregates (Nouha et al. 2018). The aggregates were carefully disrupted by a homogenizer machine to avoid cell degradation, hence the influence of the composition of the matrix. The result of the disruption is depicted in Figure 3, where the breakdown of large aggregates can be seen in comparison with the control. The relative content of individual EPS is summarized in Table 4. The homogenization step was further incorporated into a modified procedure of isolation of anammoxosomes described in this study.

**Table 4:** The relative content of individual EPS.

Compound	Relative content (%)
Saccharides	15–26
Proteins	28–35
Humic acids	18–23
Other	17–39

### 3.4 Release of the Cells From the Extracellular Matrix

On the basis of the results above, the aggregates were exposed to the hydrolytic enzymes. We started with the mixture of both Macerozyme R‐10 together with Onozuka cellulase R‐10. After 3 h of incubation on the rotary shaker and centrifugation on a Percoll® gradient, the biomass stuck on the top of the gradient (not shown here). Therefore, this action was not sufficient to release cells from the extracellular matrix.

**Figure 4:**
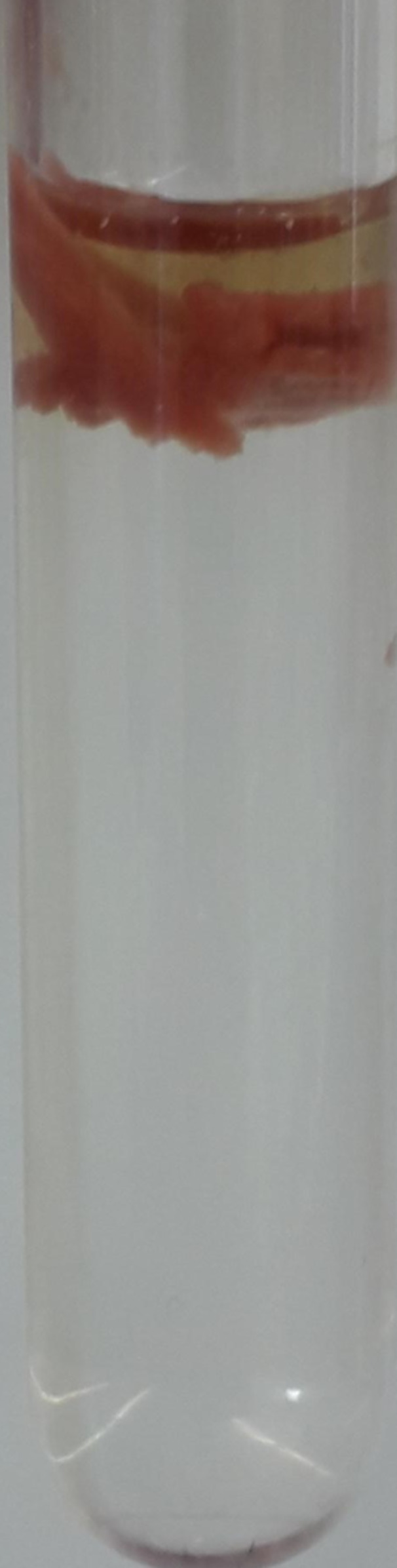
Representative illustration of separation of the cells from the aggregates. Here, EPS sample after the cleavage of EPS by a mixture of hydrolytic enzymes was applied on a 50% Percoll® gradient. The whole biomass is stuck to the top of the gradient after centrifugation.

After this, the mixture of enzymes was changed and extended with additional enzymes such as proteinase K or DNase I (see Table 2). In addition, the incubation time was extended to 24 h. After incubation, the suspension containing *Ca*. Brocadia was added to 10% to 78% Percoll® gradients followed by centrifugation. This resulted again in regluing of the biomass on the top of the gradient, as can be seen in Figure 4.

Another approach was the application of EDTA to bacterial culture. EDTA is known as one of the reagents used to extract EPS (Di Martino 2018), however, in our experiment, exactly the same result was reached by applying EDTA to the aggregated culture as well as by applying hydrolytic enzymes.

Thus, the results were the same for all experiments, suggesting that no separation of released cells occurred even on the low‐density Percoll® gradient. This suggests that either the enzymes did not sufficiently cleave the substrates, and therefore separation did not occur, or cleavage of the substrates does not lead to release of individual cells. Another possibility is that Percoll® could not be a suitable medium for the separation of such samples, so we decided to replace it with sucrose, as described in the following section.

### 3.5 Isolation of Anammoxosomes by Modified Protocol

**Figure 5:**
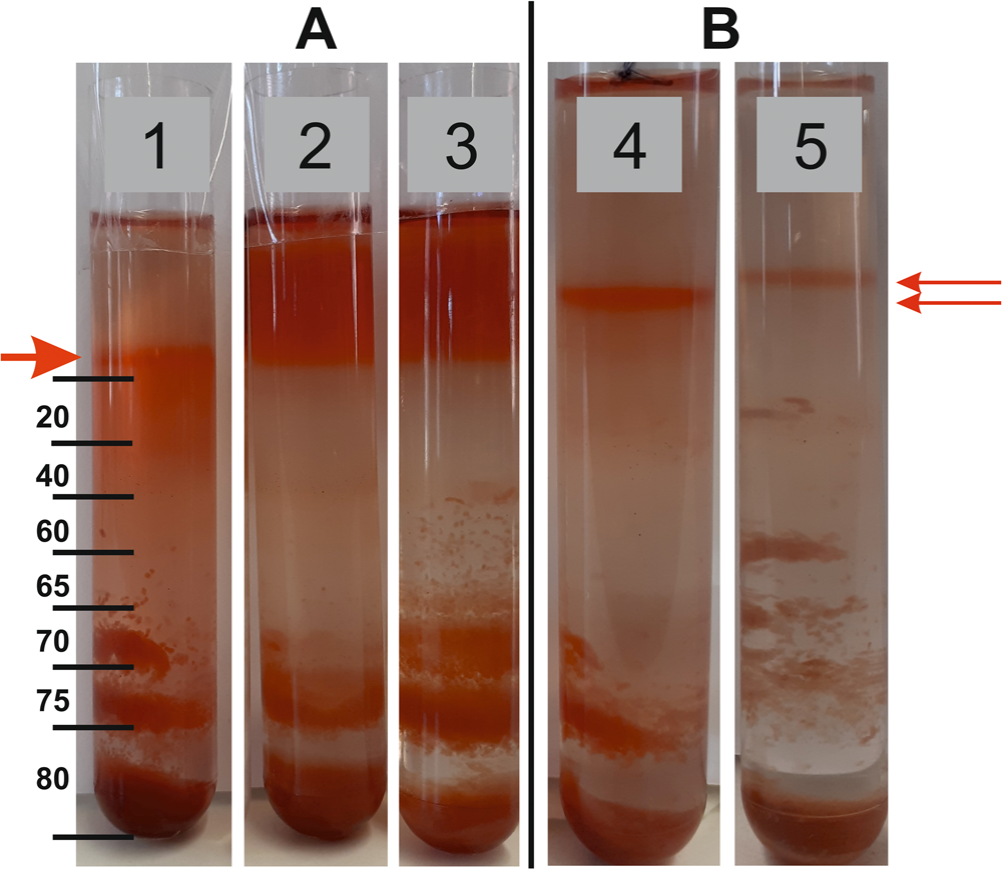
Effect of a buffer and pH on the isolation of anammoxosomes from aggregated culture of *Ca*. B. sapporoensis. (A) Isolation was performed in 1) 20 mM acetate, pH 5, 2) 20 mM TRIS‐HCl, pH 7.5 and 3) 20 mM TRIS‐HCl, pH 9. All buffers contained 60 mM EDTA. The red arrows show the position of the layers of the anammoxosome. The separation was performed on a 20‐80% sucrose (v/v) gradient. (B) The isolation procedure was repeated several times with a similar result. The images were taken under different conditions, and therefore the position of the anammoxosome layer varies.

When it proved challenging to purify anammox cells by releasing them from the aggregates, an attempt was made to directly isolate the organelles, the anammoxosomes, from the aggregated culture. To facilitate this process, sucrose was used as a separation medium instead of Percoll®, and we increased the concentration of EDTA to 60 mmol.L^−1^ by prolonging the time of incubation to 18 h. In this case, we avoid the use of hydrolytic enzymes. Consideration was also given to the impact of pH and a buffer on the isolation of anammoxosomes. Therefore, isolation was carried out in three buffers with distinct pH – 20 mM acetate buffer, pH 5 and 20 mM TRIS‐HCl pH 7.5 and 9, respectively, all containing 60 mM EDTA. In Figure 5A can be seen that the isolated anammoxosomes are located in a thin layer laying on the top of the sucrose gradient, when acetate buffer pH 5 was used. The use of TRIS‐HCl buffer together with more basic pH possibly led to the total disruption of the cells, which is indicated by the distinctive red coloring above the gradient, demonstrating soluble components inside the cells (Figure 5A, lane 2 and 3). We can conclude that the acidic pH was suitable for the isolation. It is important to note that the desired effect of EDTA was achieved by long‐term (overnight) incubation and therefore short‐term incubation could not be effective. The isolation of anammoxosomes was repeated several times, which confirmed the applicability of the method (Figure 5B, lanes 4 and 5). Finally, it is possible to compare the obtained layer with the layer from *Ca*. K. stuttgartiensis (Figure 2C) and conclude that they are very similar. In the future, this protocol could be verified for aggregates of other anammox genera, and biomasses from full‐scale wastewater treatment plants with distinct EPS content (Lotti et al. 2019). It is worth noting that *Ca*. Brocadia was previously shown to dominate in these full‐scale bioreactors (Kouba et al. 2022). In addition, the future implementation of this protocol could be attempted on already pre‐purified anammox bacteria such as *Ca*. Scalindua (Oshiki et al. 2024), *Ca*. Brocadia (Kartal et al. 2011) including *Ca*. Brocadia sinica and *Ca*. Brocadia sapporoensis, *Ca*. Jettenia and *Ca*. Kuenenia (Okabe et al. 2023).

### 3.6 Observation of Anammoxosomes by Transmission Electron Microscopy

Samples of isolated anammoxosomes from the culture of *Ca*. B. sapporoensis were fixed and embedded in resin as described in the methods. Then, ultrathin sections were prepared from the sample. The samples were contrasted and analysed by transmission electron microscopy.

In addition to the isolated fraction itself, where anammoxosomes were expected to be present, whole anammox bacteria were analysed using the procedure described above.

**Figure 6:**
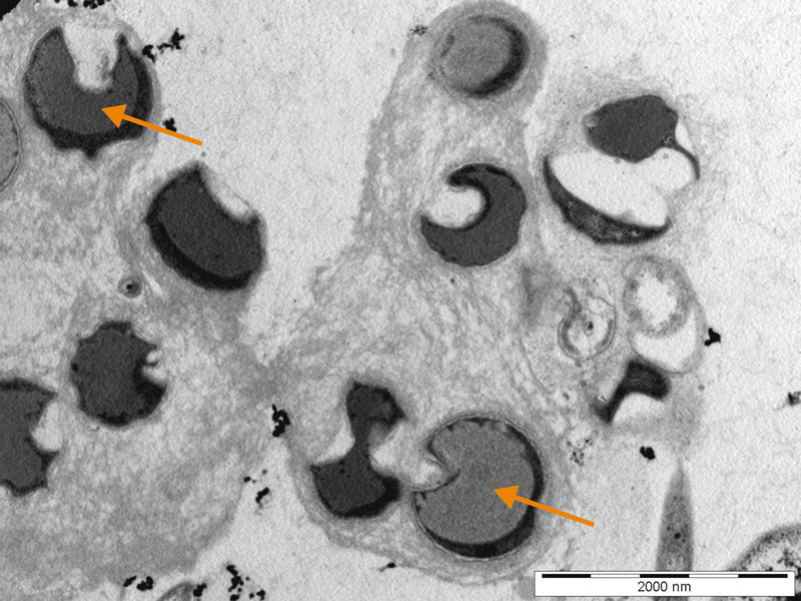
A section through intact cells of anammox bacteria observed by TEM. The anammoxosomes inside the cells are indicated by orange arrows.

**Figure 7:**
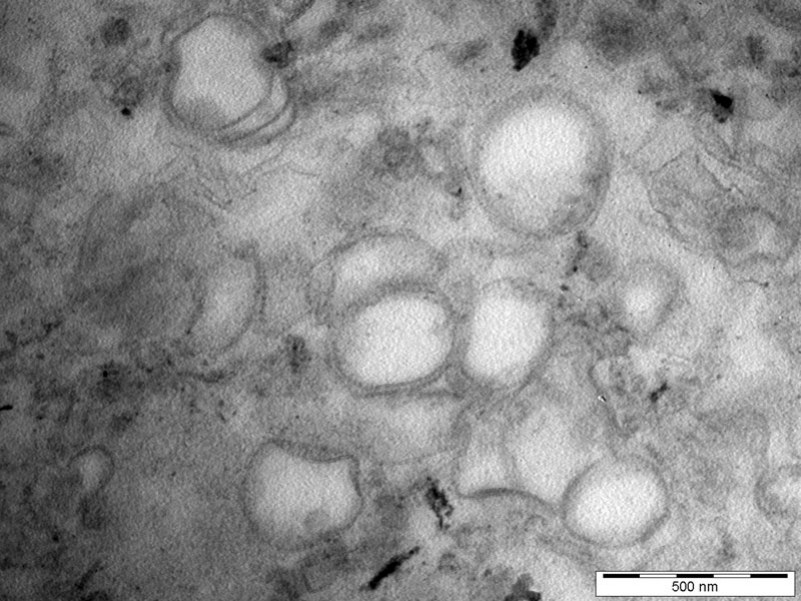
Membrane formations obtained after isolation, fixation, and contrasting of isolated anammoxosomes and observed by TEM.

A section through the bacterial cells is clearly visible in Figure 6. The characteristic structure of an anammox cell is visible in the section indicated by arrows (Van Niftrik et al. 2008; Schmid et al. 2003). The isolated anammoxosomes are visible in Figure 7.

Samples in which isolated anammoxosomes were assumed to be present contain a large number of membrane formations that do not show contrasting interiors. It is very likely that isolation has resulted in the breakage of the integrity of some of the anammoxosomes and the spilling of their contents (Ayache et al. 2010).

### 3.7 Ladderane Composition of Anammoxosome Membranes

The extracts of lyophilised mixed anammox cultures analysed on UPLC‐MS/MS revealed the content of ladderanes with C20 [3]‐ladderane on *sn‐2* and branched‐ or straight alkyl esters on *sn‐1* position (Supporting 1) with the dominant polar head groups being phosphoethanolamines (PE), then equally phosphocholines (PC) and phosphoglycerols (PG) (Supporting 2). Besides these, we detected small amounts of bi‐ladderane phospholipids, where the *sn‐2* position contained C20 [3]‐ladderane ethers and esters and *sn‐1* position contained both C20 [3]‐ladderane and C20 [5]‐ladderane. This material was further used for the preparation of the artificial liposomes.

### 3.8 Preparation of Liposomes Containing Ladderanes

To demonstrate the ability of ladderanes to be incorporated into lipid membranes, we prepared liposomes with different content of ladderane extract. Vesicles containing only extracted lipids were prepared, as well as mixed vesicles containing extracted lipids with synthetic DPPC phospholipid (see Table 3).

**Figure 8:**
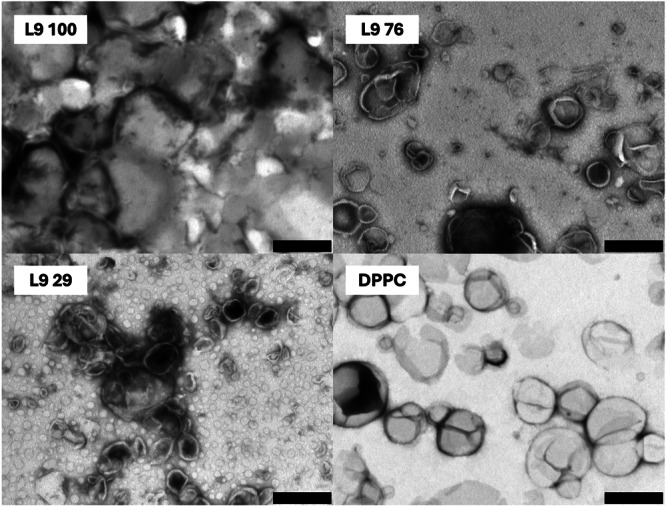
TEM micrographs of ladderane‐containing vesicles. Vesicles were prepared from mixtures of extracted lipids containing samples mixed with DPPC. Content of extracted lipids: L9 = 100%, L9 76 = 76%, L9 29 = 29%, DPPC = 0%. The scale bar corresponds to 500 nm.

**Figure 9:**
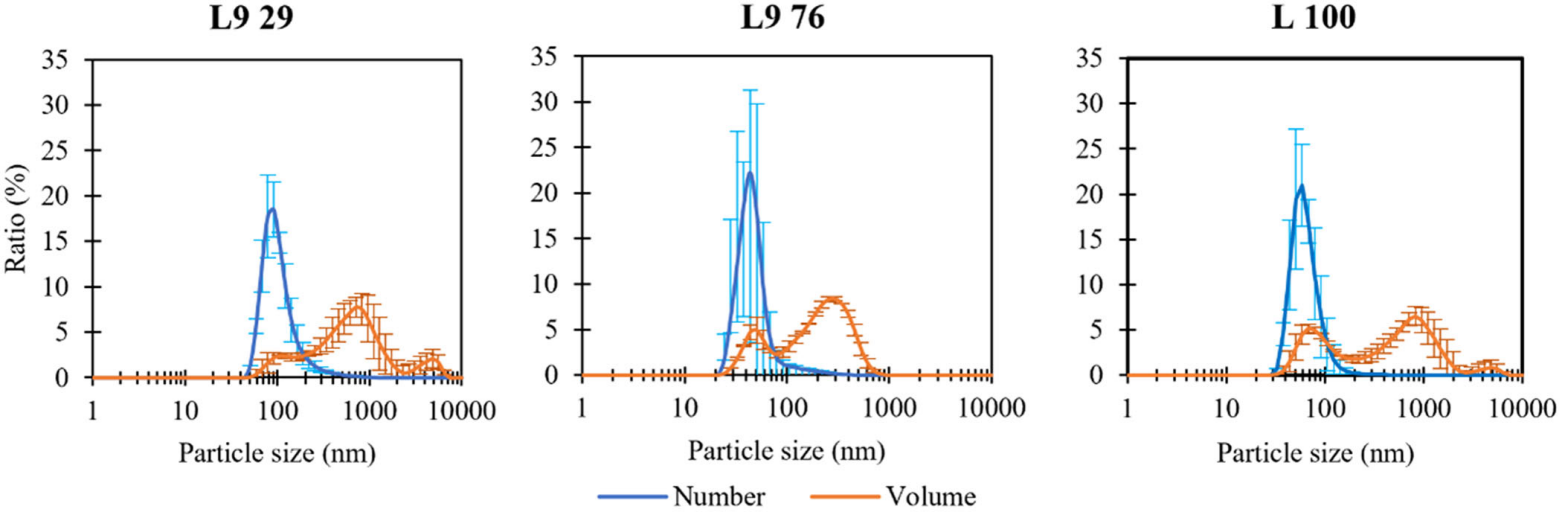
Size distributions of ladderane‐containing vesicles obtained from DLS. Vesicles were prepared from mixtures of extracted lipids containing samples mixed with DPPC. Content of extracted lipids: L9 = 100%, L9 76 = 76%, L9 29 = 29%. The error bars represent the standard deviation computed from 3 consecutive measurements of the same sample.

From the TEM micrographs (Figure 8), all samples exhibited some vesicle formation, even those without added DPPC. Decent liposomes were made for the samples containing DPPC, although there is quite a large variability in the size of the vesicles. Both bigger and smaller formations than the diameter of extrusion pores (400 nm) were created. This was also confirmed by DLS measurements (Figure 9), which showed a rather high polydispersity of the resulting vesicles. The results as such are not unexpected. The bigger particles are a consequence of extrusion membrane size, which limits the upper size of the liposomes. The smaller particles correspond to the 'natural ‐ thermodynamically stable' size of the liposomes (Pippa et al. 2014) which are always present in this type of liposome preparation.

Further vesicle characterisation was performed using CF ‐ fluorescence dye, which enables direct visualisation of vesicles in the aqueous medium. Another important feature of CF is the quenching of its fluorescence at high concentrations. Therefore, the release of CF can be directly measured using a fluorescence spectrophotometer if the encapsulated dye is concentrated enough. Carboxyfluorescein was encapsulated into the vesicles in the phase of lipidic film hydration when the CF solution was added as the hydration medium. The hydration of the film was done with TRIS‐HCl buffer instead of PBS buffer because of its suitability for work in a more basic pH area. At a higher pH, the encapsulated CF is charged, and therefore, permeation through the membrane is significantly slower.

**Figure 10:**
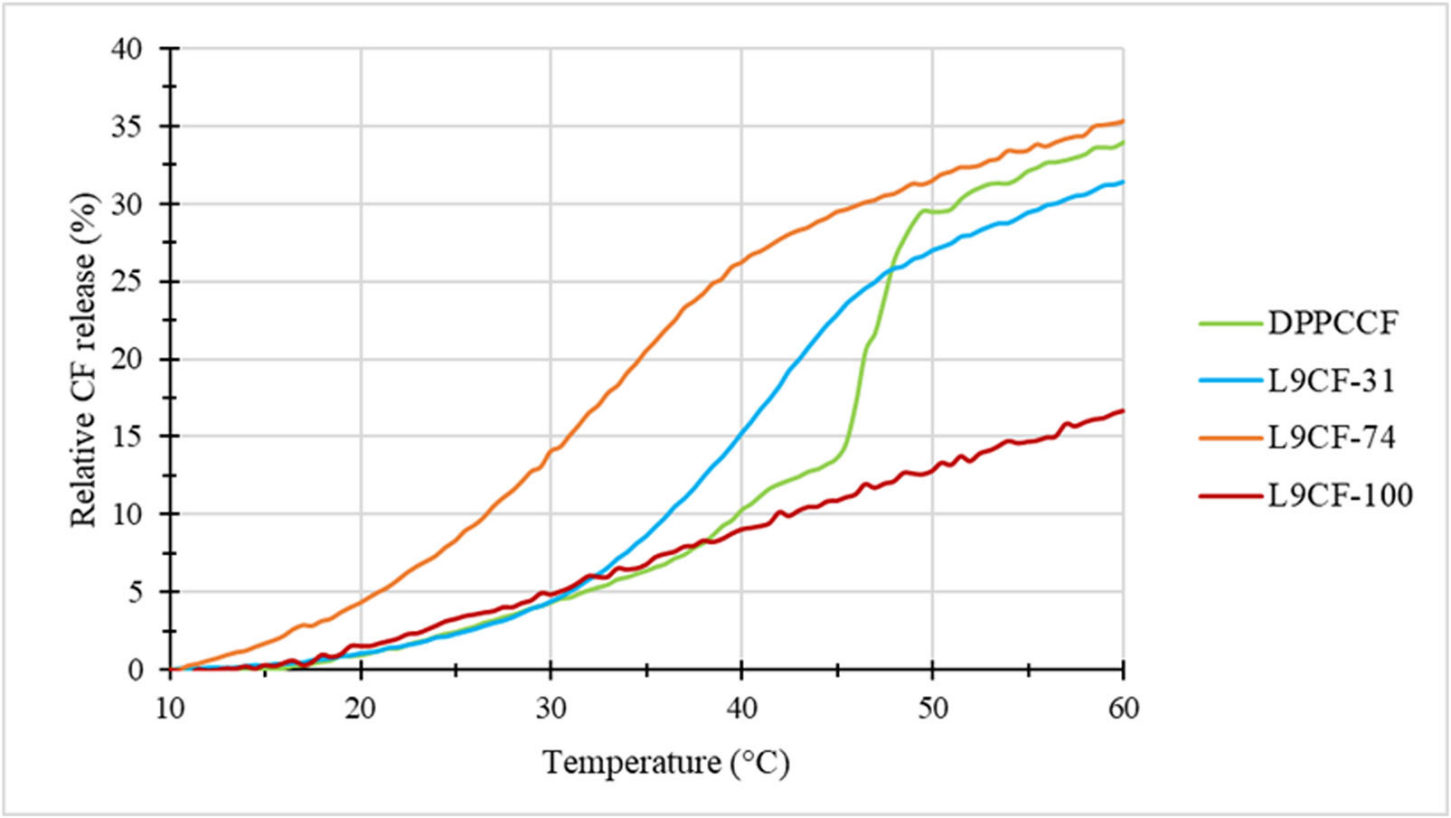
Release of carboxyfluorescein from vesicles at increasing temperature. Temperature increment 2°C per minute, fluorescence was normalised to TRITON treated sample, and the initial leaked carboxyfluorescein content was subtracted from each dependency. Content of extracted lipids: L9CF‐100 = 100%, L9CF‐74 = 74%, L9‐31 = 31%, DPPCCF = 0%.

After the separation of free CF on the prepacked chromatography column, the fluorescence of the sample was measured during a gradual increase in the sample temperature (temperature increment 2°C per minute). Data were normalised to total sample fluorescence after the addition of TRITON‐X100® for each corresponding temperature (as surfactant, it breaks the liposomal bilayer and releases the whole vesicle compartment). The relative amounts of free CF at the beginning of the experiment were subtracted from each dependency so that the temperature‐dependent sample behaviours can be reasonably compared (Figure 10).

A trend concerning the transition temperature of the membrane can be observed in the graph.

The more DPPC the membrane contains, the higher and more sigmoidal the transition is. The sigmoidal curve for DPCCCF sample shows that this membrane has a distinct phase transition temperature. As the anammox sample is a natural substrate and not a synthesized substance, it contains a variety of ladderanes and phospholipids with different phase transition temperatures. Therefore, the phase transition occurs more gradually, without a precise decisive temperature.

The decrease of the overall transition temperature with increasing anammox sample content can be caused by the low transition temperature of the contained phospholipids, i.e. ladderanes. It is even possible that the overall transition temperature for our L9‐100 sample is below 10°C, and that is why there is no transition visible on the graph for this sample.

**Figure 11:**
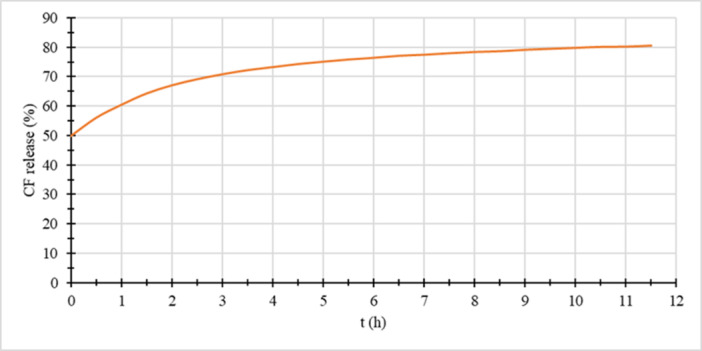
L9CF‐74 CF release was carried out at a constant temperature of 10°C.

Furthermore, the carboxyfluorescein release from sample L9CF‐74 was also measured at a constant temperature of 10°C during the time (Figure 11). Even at this temperature, the membrane is reasonably permeable for the CF present in the liposomes. Due to the material handling, 50% of the CF is already leaked even before the start of the experiment, and another 25% was released within the first 5 h. The incomplete release of CF is caused by the natural amphiphilic behaviour of the CF, which allows part of the CF to be membrane bound and thus does not contribute to the total fluorescence intensity in the measurement (Odehnalová et al. 2024). This means that particles created from the natural anammox substrate with a 26% content of DPPC (L9CF‐74) are not effectively able to keep CF encapsulated even under 10°C. The relatively low phase‐transition temperature of contained phospholipids, together with impurities of the substrate, might be the main reason for this fact. Here, it should be noted that the ladderane extract contains many different ladderanes, and therefore, some of them, if added as a pure entity, can still make the barrier properties of the lipid membrane more desirable.

**Figure 12:**
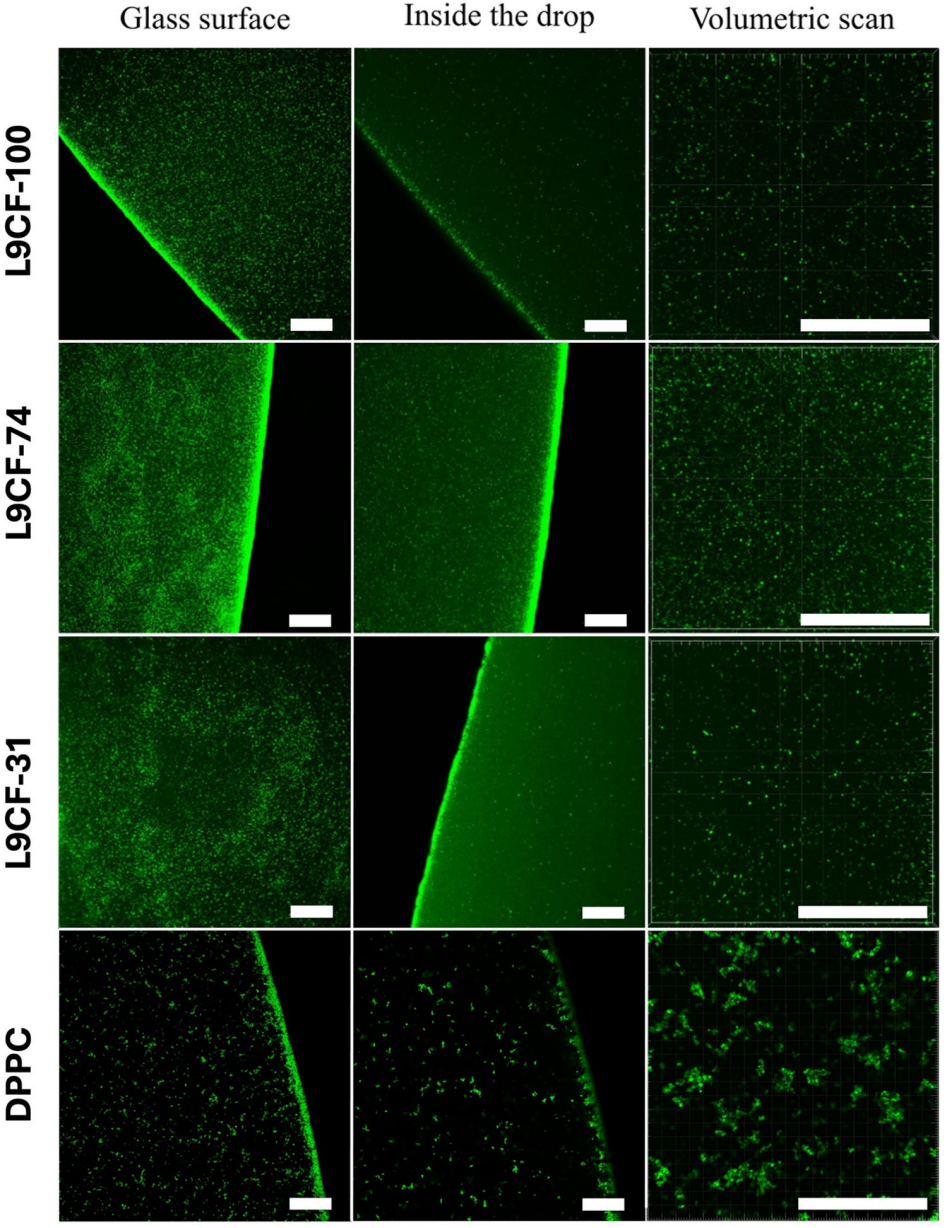
Confocal scanning microscopy of the prepared vesicles. The photos were taken from 15 µl drops in cover glass. From left to right: focus directly on the glass surface, focus inside the droplet, and zoomed in small 3D scan inside of the droplet. The scale bar corresponds to 30 µm. Content of extracted lipids: L9CF‐100 = 100%, L9CF‐74 = 74%, L9‐31 = 31%, DPPCCF = 0%.

As the last characterisation method, confocal fluorescence micrographs of samples containing ladderanes were taken (Figure 12). It is visible that none of the anammox samples present major aggregation, and individual vesicles are clearly distinguished, which is in contrast to pure DPPC sample. It seems that the addition of anammox sample prevents aggregation of lipidic vesicles compared to vesicles made of pure DPPC.

Carboxyfluorescein experiments show that addition of an anammox substrate to DPPC does not “improve” the stability of resulting vesicles in terms of their stability against leakage. However, these results prove that the substrate obtained from anammox bacteria is incorporated into the vesicle walls, since it directly influences their properties, such as resistance to leakage and aggregation behaviour.

## 4 Conclusions

This study has developed an upgraded protocol for isolation of anammoxosomes from challenging mixed and aggregated anammox cultures. Crucially, available protocols for planktonic *Ca*. K. stuttgartiensis (Neumann et al. 2014) and aggregate *Ca*. B. anammoxidans (sonication, Percoll® gradient, EDTA, sonication) did not result in the separation of anammoxosomes from debris using our *Ca*. B. sapporoensis. The upgrade consisted in omitting the prepurification of anammox cells (in comparison with the protocol for *Ca*. Kuenenia), replacing Percoll® with a sucrose gradient, and prolonging the application of EDTA. On the gradient, this yielded the layer of anammoxosomes similarly as for control planktonic biomass; the anammoxosome organelles were further confirmed by TEM. Our results strongly support the use of EDTA to disrupt the binding interactions among various EPS. Because the anammoxosome layer was localized at the top of the sucrose gradient, it is possible to omit the laborious preparation of the gradient by using only 20% sucrose; this generally simplifies the protocol. It is worth noting that during repeated isolations, it was observed that the amount of isolated anammoxosomes varied and sometimes none of them could be isolated at all. Therefore, deeper inquiry into EPS production by anammox and other bacteria could enable easier release of anammoxosomes from cells. Further, we cannot assume that this protocol can be used universally across all anammox culture/genera, as it was seen after the usage of *Ca*. Kuenenia procedure. Moreover, one attempt to apply the modified procedure to a culture enriched in *Ca*. Scalindua, the marine anammox bacteria, resulted in negative isolation (not published here). In conclusion, isolation procedure must be optimized for different either cultures or anammox genera separately.

Moreover, for the first time, we show that it is possible to prepare liposomes from anammox bacteria extract, both with and without the addition of pure phospholipids. Vesicles were successfully created, characterised by TEM and DLS, and anammox substrate was incorporated into their walls. Moreover, we demonstrate interesting functional properties of such liposomes, namely increased colloid stability at elevated concentrations. Overall, this study is an important stepping‐stone towards manufacturing ladderane‐based vesicles for drug delivery from waste anammox biomass.

## Author Contributions

**Tomáš Podzimek:** conceptualization, formal analysis, methods, investigation, writing – original draft. **Terezie Cisarová:** investigation, writing – original draft. **Michal Dvořák:** investigation. **Barbora Vokatá:** investigation, methods. **Christina Karmann:** investigation, formal analysis, methods, writing – original draft. **Jaroslav Hanuš:** investigation, writing – original draft, methods, supervision. **Martin Balouch:** investigation, methods, writing – original draft. **Matěj Malý:** investigation, methods, writing – original draft. **Jana Hajšlová:** conceptualization, writing – review and editing, supervision. **Vojtěch Kouba:** conceptualization, writing – review and editing, resources, supervision. **Jan Bartáček:** funding acquisition, conceptualization, writing – review and editing, resources, supervision. **František Štěpánek:** conceptualization, writing – review and editing, resources, supervision. **Petra Lipovová:** conceptualization, writing – review and editing, resources, supervision.

## Supporting information

Supporting information.

## Data Availability

The data that support the findings of this study are available from the corresponding author upon reasonable request.
