# Supplementary material for: Isolation of Anammoxosomes From the Aggregate Culture of Ca. Brocadia Sapporoensis and Assembly of Ladderane Liposomes
Source: Biotechnol Bioeng. 2025 May 7;122(8):2165–78. doi: 10.1002/bit.29011 (PMC12235221; doi:10.1002/bit.29011)
Supplement: Supplementary file 1 — Supporting information. [file BIT-122-2165-s001.docx]

Supplementary data

**Isolation of anammoxosomes from the aggregate culture of *Ca*. Brocadia sapporoensis and assembly of ladderane liposomes**

Tomáš Podzimek^1^, Terezie Cisarová^2^, Michal Dvořák^1^, Barbora Vokatá^1^, Christina Karmann^3^, Jaroslav Hanuš^2^, Martin Balouch^2^, Matěj Malý^4^, Jana Hajšlová^4^, Vojtěch Kouba^3^*, Jan Bartáček^3^, František Štěpánek^2^, Petra Lipovová^1^

1 University of Chemistry and Technology Prague, Department of Biochemistry and Microbiology, Prague, Czechia; tomas.podzimek@vscht.cz; Michal.Dvorak@vscht.cz; Barbora.Vokata@vscht.cz; Petra.Lipovova@vscht.cz

2 University of Chemistry and Technology Prague, Department of Chemical Engineering, Prague, Czechia; Terezie.Cisarova@vscht.cz; Jaroslav.Hanus@vscht.cz; Martin.Balouch@vscht.cz; frantisek.stepanek@vscht.cz;

3 University of Chemistry and Technology Prague, Department of Water Technology and Environmental Engineering, Prague, Czechia; Christina.Karmann@vscht.cz; koubav@vscht.cz; Jan.Bartacek@vscht.cz;

4 University of Chemistry and Technology Prague, Department of Food Analysis and Nutrition, Prague, Czechia; matej.maly@vscht.cz; Jana.Hajslova@vscht.cz;

*Corresponding author, Vojtěch Kouba, koubav@vscht.cz, University of Chemistry and Technology Prague, Department of Water Technology and Environmental Engineering, Technická 5, 166 28 Prague, Czechia


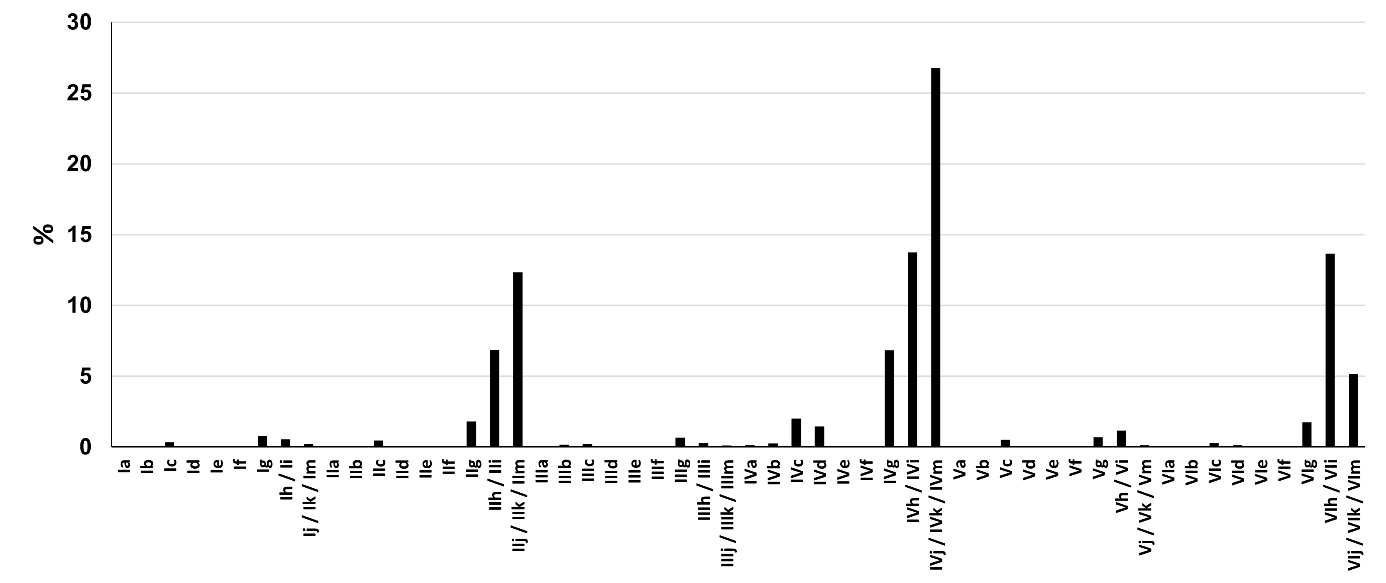


Supplement 1. The composition of detected phospholipids containing ladderane residues gained by extracting lipids from biomass. The designation of individual types of ladderane phospholipids was adopted from Boumann et al. (2009).


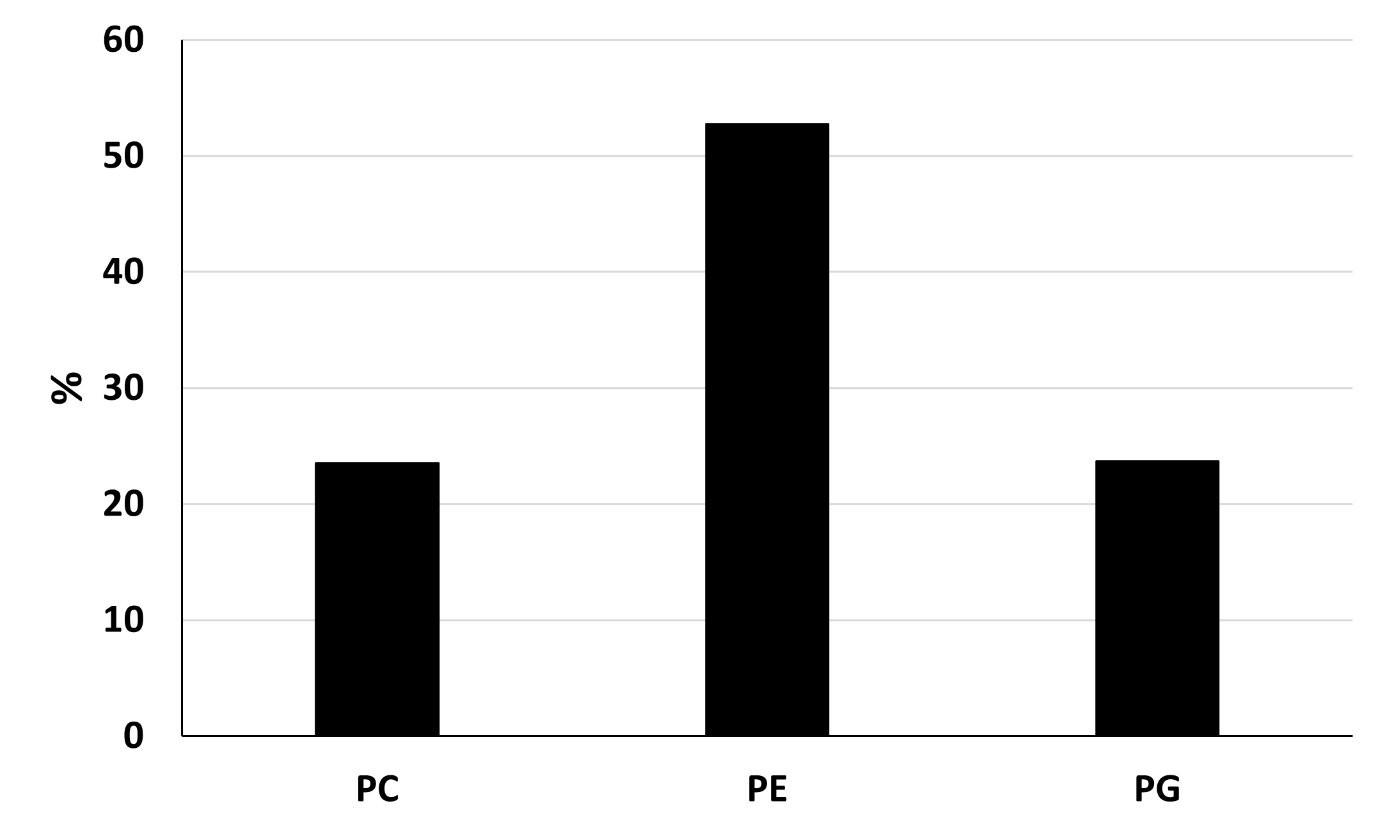


Supplement 2. Representation of individual ladderane phospholipids by polar headgroup type. PC-phosphatidylcholine, PE-phosphatidylethanolamine, PG-phosphatidylglycerol.
